# Complementary CT Coronary Angiography after Negative Selective Coronary Angiography

**DOI:** 10.5334/jbr-btr.845

**Published:** 2015-09-15

**Authors:** V. Hamoir, P. Borgies, X. Hamoir, J. Kirsch, M. Dupont, S. Kautbally

**Affiliations:** 1 Cardiology Department, Cliniques Universitaires St Luc, Brussels, Belgium; 2 Internal Medicine Department, Cliniques Universitaires St Luc, Brussels, Belgium; 3 Medical Imaging Department, C.H. Wallonie Picarde, Tournai, Belgium; 4 Department of Medical Imaging, CHU Mont Godinne, Yvoir, Belgium

A 53-year-old male was admitted for typical acute chest pain. The ECG showed a mirror image of posterior myocardial ischemia. Initial biology was normal but cardiac markers (creatine kinase and troponin) rose later. Echocardiography did not reveal any hypokinetic myocardial segment. There was no left ventricular dysfunction or valvular disease. There was no pericardial effusion or aortic dissection image. This patient was treated as a “non-ST segment elevation myocardial infarction” (NSTEMI), also called subendocardial myocardial infarction. A selective coronary angiography (SCA) was performed the next day and after careful examination by several experts, no coronary lesion was detected. Left ventriculography was also normal. Cardiac MRI was then performed and revealed a late focal subendocardial enhancement, located in the mid infero-posterior myocardial segment (Fig. A, arrow). This lesion appeared to be ischemic, despite normal SCA. Computed tomography coronary angiography (CTCA) was finally done, showing a hypodense image, with also an ischemic aspect, in the same subendocardial area (Fig. B, arrow) as observed on MRI. Furthermore, CTCA detected tight luminal narrowing with hypodense material (soft atheroma or clot) in a circumflex branch (Fig. C, arrow), corresponding to the suspected ischemic territory. In this case, CTCA both confirmed ischemic etiology and identified culprit artery missed by SCA.

**Figures A–C:**
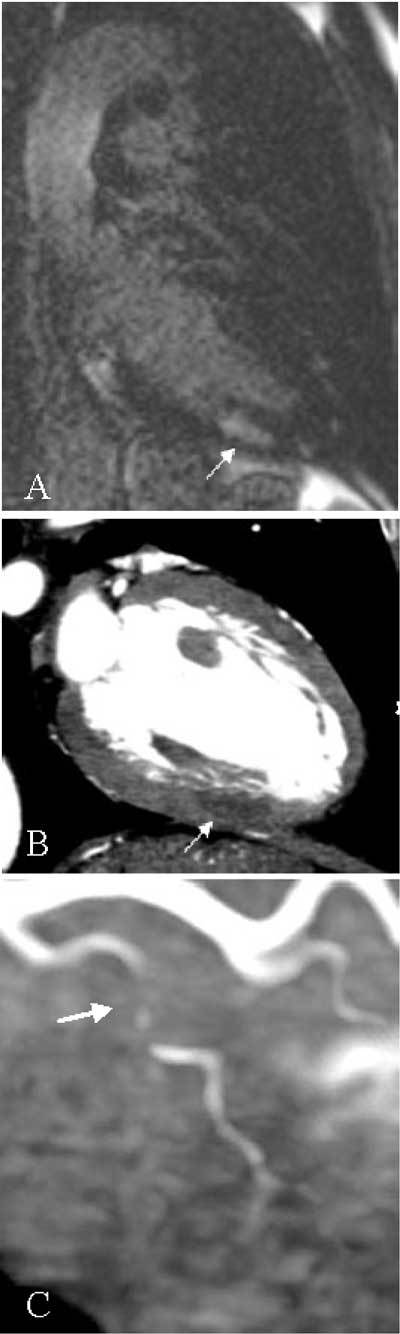


## Comment

SCA is the gold standard for investigating coronary arteries. This invasive technique gives precise information about coronary stenosis and can be immediately extended to a therapeutic approach if necessary. However, SCA can miss diagnosis in some cases such as ostial stenosis of the left main trunk, abnormal coronary origin or as demonstrated in this case. Furthermore, SCA focuses mainly on luminal narrowing but gives poor information on the arterial wall and may underestimate the importance of atherosclerosis due to remodeling phenomenon with extra luminal development of atherosclerotic plaque. CTCA is a simple noninvasive examination, becoming more performant with less irradiation thanks to modern scanners and new technical procedures such as prospective gating. It is less accurate than selective angiography for the quantification of stenosis but has a high negative predictive value. It provides information about atherosclerotic plaques including those with extra luminal development and can sometimes visualize SCA’s missed lesions. CTCA can be considered as an additional diagnostic tool in suspected ischemic patients, especially if no explanation is provided by SCA.

Regarding NSTEMI, SCA precociously performed, shows no significant coronary stenosis in approximately 10% cases with even no parietal irregularity in half of them. It sometimes demonstrates myocardial bridging, aneurysm or congenital abnormalities. However, no lesions are found in some cases even after expert analysis suggesting spasms or endothelial dysfunction hypothesis. CTCA can thus be a useful complement, occasionally bringing another explanation.spasms or endothelial dysfunction hypothesis. CTCA can thus be a useful complement, occasionally bringing another explanation.

## Competing Interests

The authors declare that they have no competing interests.
